# ‘You know you're going to get through this’: Volunteers and long‐term disaster recovery

**DOI:** 10.1111/disa.70068

**Published:** 2026-08-05

**Authors:** Nikita Sharma, Ella Kuskoff, Cameron Parsell

**Affiliations:** ^1^ Faculty of Humanities, Arts and Social Sciences and ARC Centre of Excellence for Children and Families over the Life Course The University of Queensland Australia

**Keywords:** Australia, disaster, housing, recovery, relational, support, volunteers

## Abstract

As nature‐induced disasters intensify and reliance on volunteer support grows, volunteers are now increasingly critical to disaster response and recovery, particularly in countries like Australia. This paper advances our empirical understanding of volunteer contributions to long‐term disaster recovery. The use of semi‐structured interviews with volunteers who provide disaster recovery support, stakeholders who collaborate with volunteers, and individuals who received recovery support services from volunteers allowed us to identify effective approaches that resonated with and meaningfully aided disaster‐affected people. To support individuals and families through the challenging and overwhelming recovery process, volunteers extended relational, compassionate, and agile support. By adopting a long‐term perspective focused on housing recovery, rather than engaging in a crisis response role, volunteers were able to set and manage realistic expectations concerning service delivery. Volunteers utilised both their local connections and organisational links to collaborate and communicate, confirming the need to look at the broader context within which they operate.

**Practitioner points**

- Volunteers play an integral role in long‐term disaster recovery.
- Volunteers create an inclusive, adaptable environment that responds to disaster recovery needs, especially those pertaining to housing.
- Disaster recovery support that is relational rather than transactional and individualised rather than standardised is effective for disaster‐affected people, particularly those with complex, compounded needs.
- Within the disaster recovery system, volunteers are well placed to leverage local connections and knowledge.

## 1 INTRODUCTION

Volunteers and volunteer‐led organisations are often ‘first to respond, last to leave’, making meaningful contributions to disaster response and recovery (Vallance and Carlton, 2015, p. 34; see also United Nations Office for Disaster Risk Reduction, 2015). When a disaster strikes, agencies initially operate in the response phase, focused on immediate life‐saving activities and urgent relief. Following this response and relief work, attention shifts to what is traditionally termed disaster recovery. This phase is generally defined as the ‘long‐term process of permanent reconstruction’ that unfolds over months and years (Olshansky, 2018, p. 196). For many, though, the economic, social, psychological, and physical impacts of a disaster may persist for longer, perhaps even decades (Wang and Wang, 2023; Sanderson et al., 2025b). Moreover, successive or cascading disasters extend recovery periods and compound the challenges that communities face in transitioning out of a disaster and building resilience to future events (Aldrich, 2012; Ferguson, 2023; Sanderson et al., 2025a).

Despite recovery being an essential process in the field of disaster management, there is a dearth of information on what actually works and across what time frames (Owen, 2018; Sanderson et al., 2025b). To address this lacuna, our research aims to understand what aspects of volunteer support have proved effective for disaster recovery.

We adopt a multi‐stakeholder focus that aligns with the *Sendai Framework for Disaster Risk Reduction 2015–2030*, which emphasises building knowledge through shared experiences, lessons learned, and good practices among volunteers, government officials, civil society, and communities (United Nations Office for Disaster Risk Reduction, 2015, p. 15). Within this system, formal volunteers provide vital post‐disaster support. They are unpaid individuals who have an organisational affiliation, appropriate training, or other relevant credentials. This is in contrast to informal volunteers who are unaffiliated or spontaneous volunteers who offer to help, self‐deploy, or organise themselves to assist during emergencies or disasters (Whittaker, McLennan, and Handmer, 2015). By design and through organisational positioning, formal volunteers are positioned as key knowledge holders in this ecosystem (hereafter referred to as the disaster recovery system). Yet, research has insufficiently examined their experiences and effective practices in facilitating disaster recovery. Our focus on formal volunteers addresses this gap, contributing evidence‐based insights from Australia that can inform the peer learning and knowledge sharing that the Sendai Framework advocates.

Australia serves as a critical case study for our work given that its exposure to diverse disasters triggered by natural hazards such as floods, cyclones, and bushfires has intensified significantly in recent years, creating unprecedented challenges for disaster recovery and resilience (Climate Council of Australia, 2024; Sanderson et al., 2025b). As these disasters have increased in both frequency and magnitude, volunteer contributions have grown correspondingly to meet the expanding challenges (McLennan, Whittaker, and Handmer, 2016; Grant, Hart, and Langer, 2019; Roberts, Archer, and Spencer, 2021; Phillips and Mincin, 2024).

In Australia, disaster recovery involves local communities, volunteers, community groups, business groups, non‐government organisations, and all levels of government. At the federal level, the National Emergency Management Agency is responsible for coordinating and managing national‐level emergency responses to disasters including bushfires and floods. State and territory governments have lead responsibility for on‐the‐ground response and recovery, working alongside statutory bodies such as the State Emergency Service, as well as organisations like the Australian Red Cross which provide a range of support, including emergency accommodation, food, clothing, and financial assistance. In a recent report, Sanderson et al. (2025a) discussed how non‐profit organisations are increasingly involved in providing housing recovery support following disasters in Australia. Volunteers themselves operate across a spectrum of formality, ranging from statutory roles within state and territory emergency management services, through organised and structured volunteering via non‐profit organisations, to informal and spontaneous community‐based activity. It is within this broader multi‐actor landscape that the volunteers examined in the present study operate—through an established non‐profit organisation in Queensland, Australia.

Existing research highlights the rising importance of volunteers in disaster response and recovery in Australia (McLennan, 2008; Parkin, 2008; Saaroni, 2015; McLennan, Whittaker, and Handmer, 2016). The increased contributions of volunteers align with policy expectations: the Australian *National Strategy for Disaster Resilience* recognised their valuable knowledge and capabilities, positioning them as crucial stakeholders in the disaster recovery system (Council of Australian Governments, 2011, p. 9). Yet, while the critical role of volunteers in disaster recovery is widely acknowledged, there remains limited understanding of how volunteer support specifically impacts affected communities and what elements of this support have proved effective. Given the growing reliance on volunteers and the rise in the frequency and intensity of disasters, knowing whether and, if so, how volunteer contributions actually deliver on policy expectations is vital.

## 2 VOLUNTEERS AND DISASTER RECOVERY

We begin this section by briefly reviewing the systems thinking approach, a framework that focuses on interconnected relationships and facilitates collaboration and coordination among diverse stakeholders in the disaster recovery system. The objective is to understand how volunteers are not isolated actors but function within the broader disaster recovery system. We then review the research on volunteers in disaster recovery.

### 2.1 Volunteers as system actors

Volunteer support for disaster recovery shapes and is shaped by disaster recovery systems. According to the Australian *National Strategy for Disaster Resilience* (Council of Australian Governments, 2011), disaster response and recovery responsibilities are shared among government agencies, communities, and affected individuals. It is also widely acknowledged that volunteers and nonprofits play an important part in local action, response, and recovery, as well as in advocacy (Winkworth, 2007; Akar et al., 2025; Sharma et al., 2026).

A systems thinking approach (Stroh, 2015) reveals the multifaceted impacts of disasters and the complexity of the recovery process, recognising that response and recovery emerge through dynamic interactions among multiple actors engaged in relief activities, recovery work, and collective action. This systems perspective aligns with relational approaches that emphasise how volunteers, affected communities, service providers, and government agencies continuously interact and coproduce recovery outcomes. These relationships are not static but evolve through ongoing communication, negotiation, adaptation, and learning.

Understanding these dynamic interactions is crucial for identifying potential system failures—whether through coordination breakdowns, resource misallocations, or gaps between response and recovery phases. Scholars argue that even well‐intentioned interventions can produce unintended consequences when system interdependencies are overlooked, or when institutional rigidities prevent adaptive responses (Meyer et al., 2023). By examining volunteer support through this integrated lens, we can identify effective approaches that support people in their recovery journey and anticipate and address systemic vulnerabilities that undermine recovery efforts.

### 2.2 Volunteers in disaster recovery: roles and contributions

There are a growing number of studies on disasters and healthcare volunteers (Merchant et al., 2010; Sperling and Schryen, 2022) and episodic and/or informal volunteers (Whittaker, McLennan, and Handmer, 2015; Mizui and Fujiwara, 2023; Le Dé et al., 2024); research has also looked at community preparedness and resilience building (Mutch, 2023). While these studies have increasingly highlighted volunteers' contributions to disaster response and recovery, their role and effectiveness in shaping recovery trajectories remain underexamined.

Some empirical studies analysing volunteer support for disaster recovery focus predominantly on episodic volunteers, leading to limited understanding of volunteer roles beyond the immediate disaster response phase (Sauer et al., 2014; Daddoust et al., 2021). Others focus on volunteer behaviour and turnout after disasters. For example, while Iizuka and Aldrich (2022, p. 527) emphasised that volunteers provide ‘resources, emotional support, and labour at a time when local responders are often overwhelmed’, their article centred on volunteer turnout and not the impact on and effectiveness of local communities. Similarly, Acosta and Chandra (2013) discussed how cultural and faith‐based organisations provide support services through philanthropy and volunteer management as well as spiritual and emotional care in the aftermath of a disaster. Although the presence of volunteers during the response phase, which occurs immediately after a disaster strikes, is well documented and has received significant research attention, the sustained contributions of formal volunteers, who continue working with affected communities well beyond the initial emergency response, are overlooked in this strand of work.

Research highlights that volunteers and volunteer‐led organisations play a multifaceted role in long‐term disaster recovery, extending well beyond physical rebuilding to include case management, transitional housing support, and addressing the needs of vulnerable populations through inter‐organisational collaboration (Phillips, 2014). Experienced volunteer‐led and non‐profit organisations are particularly valuable in coordinating skilled and low‐skilled volunteers, navigating bureaucratic processes, and filling gaps left by formal recovery systems (Phillips and Mincin, 2024). Akar et al. (2025) underscored that volunteers make unique relational and creative contributions to disaster recovery that complement but extend beyond professional practice.

Recent comparative studies by Akiyama (2019) and Park and Yoon (2022) reviewed recovery trajectories in China and Japan and in South Korea and Japan, respectively. These works reveal that civil society actors, especially volunteers, contribute to disaster recovery in Japan and South Korea through speed and flexibility to fulfil unmet needs, particularly those of vulnerable populations. South Korea, however, has a less structured volunteer‐driven response than Japan. An important insight from these studies is that locally embedded organisations demonstrated greater staying power because their staff and volunteers lived in affected areas and had a long‐term commitment to community recovery. Akiyama (2026) revealed that the non‐profit sector in Japan utilised its networks and social capital to support disaster recovery efforts after the Tōhoku earthquake and tsunami in 2011. These studies also show that volunteers and the non‐profit sector mainly contribute to the socio‐emotional recovery of those affected by disasters. Collectively, they underline the merits of locally embedded support for disaster recovery, qualities that have been observed and documented in recent decades.

In the Australian context, however, the contribution of nonprofits and volunteers in disaster recovery reflects a far longer and more deeply institutionalised tradition. For instance, the literature on volunteers from Japan further highlights how they have become an indispensable part of the disaster response and recovery process since the Kobe earthquake in 1995 (Iizuka and Aldrich, 2022). This is in contrast to Australia's more established volunteering and non‐profit sector and civic‐driven culture of participation, particularly in the area of disaster recovery (McLennan, Whittaker, and Handmer, 2016; Innes, Jefferies, and Gates, 2025). Australia's volunteering infrastructure promotes and advocates volunteering efforts and the *National Strategy for Disaster Resilience* (Council of Australian Governments, 2011) acknowledges the fundamental role of volunteers in disaster recovery in the country. Nonetheless, there remains a significant empirical gap in the literature on how volunteers more broadly support disaster‐affected people.

Meyer et al. (2023) studied long‐term recovery groups that focus on disaster case management to address unmet needs. In comparison to research that looks at volunteer roles in disaster response or early recovery tasks, these authors focused on groups that prioritise a range of long‐term household recovery needs, such as employment, housing, and childcare. These groups, through volunteer coordination and donated resources, may either complement or supplement the actions of government and other recovery support service providers. Meyer et al. (2023) concluded that there is a need for a better understanding of their actual impact on recovery and operational effectiveness. We aim to fill this gap in knowledge by studying the work of volunteers involved in long‐term disaster recovery support.

Research on disaster recovery and management support provided by social workers also emphasises the disaster recovery needs of marginalised subgroups (Dominelli, 2015; Alston and Chow, 2021; Hay and Pascoe, 2021, 2022). While social workers' contributions to long‐term recovery are well documented in the literature, including their effect and the challenges confronted, less is known about the impact of volunteers who often operate with different resource constraints and capacities.

Few studies highlight the growing importance of voluntary efforts in disaster response and recovery and ultimately resilience (McLennan, 2008; Parkin, 2008). And even when they acknowledge the complex interdependencies that characterise disaster management networks in countries like Australia (McLennan, Whittaker, and Handmer, 2016), their focus is largely on strengthening the capacity of volunteers and the non‐profit sector.

The risk of disasters is increasing in Australia, and this translates into greater and diverse recovery demands, as communities encounter longer, more complex paths to rebuilding after each event (Rice et al., 2022; Sharma et al., 2026). Existing Australian research prioritises disaster response and preparedness over recovery (Botterill and Wilhite, 2005; Winkworth, 2007; Oloruntoba, Sridharan, and Davison, 2018; Atkinson and Curnin, 2020). However, scholarly attention to volunteer contributions in disaster contexts has grown. Where volunteers are examined, research has noted organisational challenges such as recruitment and retention (McLennan, 2008) and the evolution towards more diverse, community‐based forms of engagement (McLennan, Whittaker, and Handmer, 2016). For example, McLennan (2008) underscored challenges around organisational governance and the sustainability of volunteer‐based models in the face of changing community expectations and operational demands following disasters.

Roberts, Archer, and Spencer (2021) revealed how non‐profit organisations, and specifically motivated volunteers, can support affected communities in recovering from disasters. Their research also emphasised the importance of community connections and networks in fostering resilience against future disasters. Similarly, Serrao‐Neumann, Crick, and Low Choy (2018) argued that stronger engagement between volunteer‐based recovery agencies and communities can harness local knowledge and personal networks to enhance service delivery.

Collectively, the literature has identified various strengths of volunteers in the post‐disaster setting, including leveraging community access, and local knowledge, understanding vulnerability, and providing information and support services. Despite research that has documented the valuable contributions of volunteers in the immediate aftermath of disasters and during response activities, there is a need to analyse the approaches they adopt to support disaster recovery.

## 3 METHODS

Our research used a qualitative approach to examine empirically volunteers' experiences of supporting individuals and families affected by nature‐induced disasters, as well as disaster‐affected people's experiences of receiving volunteer support. The main aim is to understand what approaches have proved effective for volunteers in supporting long‐term disaster recovery.

The support providers who are the focus of this study volunteer through an established nonprofit in Queensland, Australia. This organisation offers multiple support services and initiatives including disaster recovery support, financial hardship assistance, and education and employability programmes.

The study involved in‐depth, semi‐structured interviews with three core cohorts. The first cohort we interviewed was volunteers contributing to the aforementioned non‐profit organisation in Queensland, Australia (n = 12). Of the 12 volunteers, five were women. All volunteers are local and embedded in grassroots‐level groups of the nonprofit and are experienced in leveraging established relationships and networks to foster connections with disaster‐affected people. The nonprofit has several supportive and protective resources and measures in place, including a dedicated manager for oversight, training, and accountability, paired working arrangements to reduce isolation and vicarious trauma, and safeguarding training. Volunteers also operate within a clearly defined scope. Specifically, these volunteers provided disaster recovery support to those affected by various nature‐induced disasters in Queensland between 2022 and 2024, including the floods in southeast Queensland in 2022, the Western Downs bushfires in 2023, and Tropical Cyclone Jasper in the far north of Queensland in 2023. Together, these disasters resulted in billions of dollars of social, economic and health costs across the state.^1^ The scale and period of disaster recovery work varied for each event, with support coming from a variety of local community groups, stakeholders, and councils.

The second cohort we interviewed was disaster‐affected individuals who received support from these volunteers between 2022 and 2024 (n = 11). These interviews sought to gain insights into individuals' experiences of receiving support, including firsthand accounts of the support extended and which forms of support were found to be most valuable. In recognition of their contribution to the research, disaster‐affected individuals who participated in an interview received an AUD 50 gift voucher.

The third cohort we interviewed was stakeholders who supported the volunteers in their delivery of the disaster recovery support services or collaborated with them to support impacted communities (n = 4). These people have experience of disaster response management, governance, and coordination. Including stakeholders in this research enabled us to understand how volunteers engage and collaborate with others involved in recovery support delivery and operate within the broader disaster recovery system in Queensland.

Ethical approval for this study was obtained from The University of Queensland (2024/HE000152). Semi‐structured, qualitative, one‐on‐one interviews were conducted between May 2024 and June 2025 and usually lasted between 30 and 90 minutes. Owing to the geographical spread of the participants, interviews were conducted via telephone. A series of guiding questions were used to gather rich information on participants' insights on what worked well, for whom, what did not work as intended, and why or in what circumstances.

With participants' prior informed consent, interviews were audio recorded and transcribed verbatim. Drawing on a thematic analysis approach (Braun and Clarke, 2006), the lead author, Nikita Sharma, began by becoming familiar with the transcript data. Informed by this data, as well as the key aim of the study, we abductively developed a coding structure. Then, using NVivo software as a data management tool, the lead author coded the transcripts, engaging in frequent collaborative discussions with the other authors throughout the process. The codes were then arranged and grouped into analytic themes; the most prominent of them were selected for inclusion and discussion in the findings below. Given the relatively small sample size and the importance of maintaining anonymity, we have chosen not to add participant numbers or potentially identifiable demographic information when presenting quotes throughout our findings.

## 4 FINDINGS

This section is organised around the key themes we identified in the research participants' accounts of providing and receiving disaster recovery support. We also incorporated stakeholders' accounts to gain a deeper understanding of the volunteers' approach to supporting people's disaster recovery journey.

The volunteers' approach to supporting disaster‐affected people encompassed three interconnected elements. First, they adopted a long‐term perspective centred on housing recovery. Second, they provided individualised, people‐centred support grounded in relationship building. Third, they leveraged community connections and local networks to access resources and navigate recovery systems.

### 4.1 Taking a long‐term view to guide housing recovery

Volunteers consistently described their work as extending well beyond immediate disaster relief to entail long‐term recovery support, with a particular focus on housing recovery. Rather than acting as crisis responders, the volunteers positioned themselves as sustained, complex support providers. In interviews, volunteers explained that their primary objective was often to support people with housing recovery. To achieve this goal, volunteers deliberately avoided taking on the role of the first responder, which is typically performed by the emergency services. Although at some level it sounds counterintuitive that these volunteers refrain from supplying immediate and urgent support, but they elucidated that this is a strategic approach that takes into account their learnings from providing disaster recovery support. As one volunteer explained:

*We're not first responders, and we really, we try and make it very clear that we're not first responders, and we stay away from them because they've [first responders] got a difficult enough job as it is without us jumping in there to interfere, so to speak, or get in the way*.

Positioned outside of first response, the volunteers discovered through practice and experience that sustained engagement drives housing recovery. They further pointed out that adopting a long‐term recovery perspective, rather than assuming a crisis response role, was instrumental in establishing and managing realistic expectations around service delivery.

The volunteers in this study supported disaster‐affected households through the multifaceted process of housing recovery. This included assistance with dwelling repairs and reconstruction, as well as helping people to replace essential household goods and furniture damaged or lost during a disaster.

This experience taught volunteers to anticipate the extended timeline of housing recovery, allowing them to position themselves strategically in cases where long‐term needs emerged and other services had been withdrawn. One of the volunteers aptly summed up the nature and timing of demand for housing repairs post disaster:

*Just going on memory, some people take up to 12 months or possibly more to get to the point where they're looking at doing those repairs, so they're not in the state of mind. It's [that] the problem in front of them is just so onerous. It takes that long. They've been through counselling. Now they're at a point, okay, I'm ready to face the world again. Oh, but I've got no money, and that's where we can step in as well. That's where a lot of times we do step in*.

Prolonged engagement is important to address evolving needs as recovery time frames may amount to up to a couple of years for some. The intensity of personal experiences, as well as the duration and frequency of support services, varied among the disaster‐affected individuals who participated in this study. This is not surprising as personal experiences are likely influenced by the magnitude and severity of the disaster event. Our research participants also discussed how housing recovery is a lengthy and time‐consuming process. For example, 2.5 years after the floods in southeast Queensland, one of the disaster‐affected participants stated that they were still working towards full housing recovery:

*No, the work never finished. 2022 flood project is still not finished yet. After [the] flood, we've not gone to the house*.

The experiences of disaster‐affected people reveal that these volunteers filled service gaps often left by market mechanisms such as insurance or public service instruments. Both volunteers and affected individuals highlighted the prevalence of and challenges posed by inadequate insurance coverage, whether through underinsurance or a complete lack of insurance. In such cases, volunteers played a crucial role in housing recovery by filling support service gaps. A disaster‐affected participant said:

*But they knew that we were in a situation where we couldn't even afford [the work]. There was no insurance on the house, and we couldn't afford to get the roof fixed, because it was a full replacement of a roof*.

Paralleling the experience of those affected by disaster, volunteers also described housing recovery support for homeowners and renters. Such work encompassed both practical assistance with replacing lost household goods to coordinating major structural repairs, as well as administrative support with navigating the paperwork required for repair quotes and funding applications. One volunteer captured this breadth of activity:

*Like some people, you know, needed some structural repairs, but mainly they needed replacing goods, you know, furniture and goods that they'd lost. Other people, their houses needed to be gutted because they don't make houses with wooden walls anymore. They use that stuff that melts away. A lot of walls needed to come out, and they needed to be redone. So, we get people to get quotes for their repairs, and we had all sorts of forms they had to attach*.

Others also shared how they navigated multiple challenges related to the support of repair and reconstruction work, including coordinating with tradespeople and builders and dealing with paperwork and other bureaucratic obstacles. As one volunteer put it:

*Some people, you know, we'd do all the other paperwork, and then getting the quotes into us was delayed. They couldn't get people to come and give quotes*.

Volunteers also described navigating the practicalities of providing housing recovery support, including sensitivity to distressed individuals. Their insights reflect what the human dimension of housing recovery support looks like. One volunteer reflected on how they manage bureaucratic and practical tensions, describing the difficulty of asking disaster‐affected people for ‘paperwork’ while sitting in their damaged homes:

*When you're asking people for paperwork, it felt a little bit hard, but we tried to be as reasonable as we could … getting quotes was the thing that was stressing some people, because they're thinking, well, no one wants to come and give it … how do we know a builder we can trust? And people were just so grateful that someone came and saw them at home, or in the wreck of [a] home in a lot of cases*.

Volunteers' housing recovery support thus prioritises reconstruction, definitive repairs, and the restoration of safe and liveable conditions. Volunteers' accounts and the experiences of disaster‐affected people also provide a better understanding of the nature and duration of support that leads to housing recovery. They also reflect the quality of voluntary commitment and the distinctly human dimension of volunteer‐led housing recovery.

### 4.2 Offering relational and individualised support

A foundational element of volunteer support is an assessment of the disaster impacts on the individuals and households that seek assistance. Volunteers conduct these appraisals to understand not only physical damage but also the specific issues pertaining to circumstances, resources, and challenges that each household faces in recovery. While such assessments often involved home visits, volunteers frequently gave affected individuals the option to meet elsewhere if they were uncomfortable with such an arrangement owing to disaster‐related damage. One volunteer emphasised:

*The disaster recovery area, how they get the people who go out and do assessments, and things like that, it makes such a difference. Like, you know, you get a much clearer picture of how you can help people and then speak with them. And I must say, all of the interviews which we've done, we have never had, that I'm aware of anyway, we've never had anybody be critical of them. So, they must be doing something right*.

While volunteers prioritise an assessment to gauge what support is needed, they approach it with understanding and compassion, as well as engaging in active listening—a relational style that itself facilitates the long‐term recovery process by building the trust necessary for effective support. This compassionate stance encourages those affected to open up about their losses while receiving emotional support during conversations with volunteers. The assessment process itself is conversational and empathetic, tailored to the unique situation of affected individuals and households. Through this approach, the assessment transcends its traditional role of simply identifying damage and recovery needs and becomes the foundation for building meaningful relationships that sustain people in their long‐term recovery journey.

Another volunteer explained how their relational approach provides much‐needed socio‐emotional support to those affected and instils a renewed belief in their ability to move forward:

*People don't feel empowered. You need to empower people after a situation like this. You need to build up their confidence. Everyone is entitled to be treated with respect. And it's almost like having a dig; while you are down and out [others] will give you shit stuff, people don't want that*.

A disaster‐affected participant described volunteers as akin to (if not better than) professionals in the disaster recovery space. The following remarks by two disaster‐affected persons illustrate how these volunteers uphold professional standards and deliver quality services even in high‐need, high‐stress situations:

*The only people I've had really good help and professional help, and are very, very good is [the volunteers]*.

*Anyhow, dealing with [the volunteers] was a humanising experience in a sea of insensitivity and hoop jumping*.

Interviewees also described how volunteers' professional and compassionate approach had a positive impact on their recovery process. Furthermore, having familiar, consistent engagement with the same volunteers provides psychological comfort and stability while fostering trust between volunteers and affected individuals during the chaotic, extended recovery process. It also allows volunteers to adjust their assistance as recovery needs shift over time. In the words of a disaster‐affected participant:

*They were so lovely, so nice, and, and they sat in [my damaged home] and then we worked out a system of ongoing communication, and she [the volunteer] would ring me up and I would be distracted, and they would never judge…. They were just really; I don't know the word: non‐judgmental…. They didn't make you feel worse or bad, for not performing and not getting those things done on time…. And what was good is they had the same person that I contacted each time, and she knew my story too, so then I didn't have to repeat myself*.

The disaster recovery process is not only a function of the support received but also of people's capacity to cope with the impact of a disaster, often measured in socio‐economic terms (Hallegatte et al., 2017). This variability was evident in the diverse population served by volunteers in this research, including people of different ages and with different socio‐economic backgrounds and abilities.

Participants' experiences revealed how disaster impacts are compounded among those facing multiple vulnerabilities: young families, renters, precarious workers, pensioners, and people with disabilities and long‐term health conditions experienced disproportionate challenges that required tailored, responsive support. These characteristics often signal underlying socio‐economic vulnerabilities, which differentially expose individuals to heightened risk following adverse life events such as a disaster. An interviewee who received support shared that young families may need school supplies to avoid the follow‐on impact of a disaster on children's schooling and education. Additionally, renters may have to relocate urgently if the rental market becomes increasingly competitive after a disaster. One of the stakeholders who participated in this research underlined that vulnerabilities manifest in multiple forms, such as with respect to financial precarity, cultural and linguistic background, digital divide, and geographic remoteness. These dimensions influence not only how severely people experience disasters impacts, but also their resources and capacity for recovery. The stakeholder added:

*So, people from [the] lowest, lower socio‐economic group are disadvantaged. People who don't have good technology skills, they're very disadvantaged. Those that don't have an e‐mail, those that don't have a computer, those who aren't good with mobile phones or don't have a mobile phone, they're very disadvantaged because most of the government things are now all pushed towards people who have a mobile and people [who] have an e‐mail…. There are different groups, such as the ATSI [Aboriginal and Torres Strait Islander] community, disadvantaged, and also from a culturally and linguistically diverse background, [that] could be especially [impacted] if they haven't got English as their first language. That makes it hard, because they're trying to learn some of the terms with the government departments…. These are the most vulnerable groups that I can see*.

These various forms of disadvantage often compound one another, creating unique recovery challenges that demand equally varied support. In one instance, a volunteer shared how they purchased a tablet computer to support an autistic child in the care of a grandparent during the 2022 floods in southeast Queensland. The family, which was renting at the time, had been directly affected by the disaster. Amid the overwhelming tasks of salvaging belongings and finding temporary shelter, the grandparent worried most about the child's distress at losing their routines and familiar surroundings, underlining how maintaining connection and a sense of regularity is critical for disaster recovery.

Such individualised support stems from the relational approach that these volunteers pursue. The experiences of participants who received support services highlight how volunteers provide socio‐emotional support that is grounded not in protocol, but in empathy and attentive listening. The relational support of volunteers, strengthened by repeat visits and a willingness to see each affected household as unique, enables them to identify and address diverse recovery needs.

### 4.3 Leveraging community connections and networks

The interviews revealed that one of the key attributes of volunteers is localised support and processes to enable disaster recovery. The volunteers in our study belong to grassroots groups that function within an established nonprofit, allowing them to leverage both their local knowledge and community connections. Localised support was also a factor in significant work by the volunteers in collaboration with other stakeholders and key coordinators, as well as in communicating with employees at the non‐profit organisation. One volunteer summed up this aspect as follows:

*That coordination role and communicating with other agencies and charities has been a huge job*.

Their responsibilities included data entry and processing and filtering and communicating crucial information on disaster impacts and recovery needs. This was facilitated by their ability to leverage their local community networks and their understanding of the disaster recovery management and governance framework in Queensland.

On the one hand, the volunteers leverage their local networks and connections within affected communities to learn about disaster impacts and support those involved. In the aftermath of the floods in southeast Queensland, volunteers worked in close collaboration with local community actors from neighbourhood centres and schools, which led to greater situational awareness and increased community outreach. On the other hand, the volunteers also establish and maintain collaborative relationships with other community organisations or government agencies to minimise support service delivery gaps. While their local knowledge provides them with greater situational awareness, this does not mean that volunteers work in isolation. Instead, they use this to strengthen the disaster recovery system. A stakeholder emphasised volunteers' commitment to minimising service gaps:

*So, the good thing I found is that [the volunteers] are very collaborative with other organisations. It's not a matter of saying, no, we want ours to be the only service in town. It's a matter of saying, well, we're trying our best to help out the people, regardless of where they are within the state, and regardless of who else might be able to help them as well*.

While the main coordination role is often shouldered by government agencies, the focus on localised support means that volunteers have often assumed coordinating roles on a smaller but meaningful scale. One volunteer recounted their experience of working closely with local organisations and reflected on their need to leverage further opportunities for local collaboration:

*We sort of set about a small committee which comprised people from our school, school communities, as well as from our organisation and our civil conference. So, we've set up a little group. If we have another disaster, then contact this person from here. But that's within our own community, really. But we've also got another organisation across the road, and we've got contacts with them, so we're doing it in a very small way, but that's just locally…. So, talking to whether it be schools or other community agencies, to sort of say: ‘Hey, look, we may be able to help with this, can you help with that sort of thing?’*

Since these volunteers are embedded within local communities, their local knowledge and community connections help them to better understand the impact of a disaster. This assists with gathering local information that is often preferred over broader‐scale knowledge in such settings, as the former provides granular insights to support the recovery of individuals and households (Taylor et al., 2023).

Embedded volunteers benefit from pre‐existing trust within their communities. They understand local needs, social networks, cultural norms, and the pre‐existing vulnerabilities that shape how disasters affect different populations. Unlike external disaster relief and recovery organisations that deploy temporarily and then withdraw, community embedded volunteers remain present throughout the long‐term recovery process. This allows them to build sustained ties and relationships that serve as investments in community recovery. A stakeholder in the disaster recovery system also noted the benefits of engaging community embedded volunteers in the provision of disaster recovery support services:

*Their willingness to jump in and support is obviously well received. There are limited roadblocks when you're trying to engage those agencies. So, they're more agile than larger organisations with really structured governance processes; that's probably what works well: the ability to mobilise people who have got the right attitude, the right approach, and who are very passionate about what they do. You know, that goes a really long way*.

The above account highlights how agility is a hallmark of the support of volunteers. This is evident in their rapid coordination and flexible service delivery, both of which are enabled by strong local partnerships. A stakeholder shared how agility combined with passion make these volunteers essential and highly effective partners in disaster recovery. As discussed earlier, they often fill gaps in disaster recovery support service delivery, yet their role extends well beyond meeting residual needs. Unburdened by complex governance processes and bureaucratic constraints, volunteers are able to mobilise rapidly and supply invaluable recovery assistance.

Effective disaster recovery requires robust communication, coordination, and collaboration with actors in the disaster recovery system. As such, coordination and collaboration between various entities are vital for operational efficiency and providing comprehensive support to aid recovery. Volunteers who directly engage with disaster‐affected individuals are uniquely positioned to identify emerging trends, resource shortfalls, and unanticipated needs that may not be visible from an organisational or higher level. Their engagement is likely to result in resources being allocated more effectively and responses to changing conditions in real time. Volunteers also help with the flow of information from the organisational to the individual level. Given the embeddedness of volunteers within affected communities, their communication and coordination efforts can help to avoid gaps in service coverage, minimise duplication of efforts, and enable a rapid response to support the closure of gaps, whether existing or emerging.

## 5 DISCUSSION

Volunteers play an important role in disaster response and recovery. These overlapping phases and the lack of clarity on relief, response, and recovery time frames reflect a problem: there is little empirical evidence regarding what recovery interventions actually work or over what duration (Owen, 2018; Sanderson et al., 2025b). Our research interrogates what makes volunteer support effective in disaster recovery by examining the experiences and practices of formal volunteers.

The findings of this study show how volunteers support affected people in recovering from disasters in the long run. While the current literature is skewed towards the role of episodic or spontaneous volunteers or those involved in emergency assistance, the volunteers in this research often provide much‐needed support during the extended recovery phase. This research highlights the complex, ongoing work performed by formal volunteers who are integral to comprehensive recovery efforts in Queensland, Australia.

The findings deepen our understanding of the impact of volunteers' efforts on disaster recovery that are of longstanding interest to policymakers and researchers. The focus of volunteers on housing recovery reflects legitimate differences in priorities: while emergency service volunteers concentrate on immediate relief and response, and spontaneous volunteers' activities centre on initial support, the volunteers in this study address housing needs that propel disaster recovery.

Our study extends recent research that focuses on psychological and emotional recovery as the main contribution of volunteers and nonprofits (Park and Yoon, 2022), demonstrating that formal volunteers provide essential emotional and material recovery support. This is not surprising as local governments are frequently overwhelmed and lack the resources to meet community housing needs during the disaster recovery phase, including significant backlogs in the processing of planning and building permits for dwelling repairs and replacement (Sanderson et al., 2025a). This is particularly important as housing recovery is widely recognised as a vital and complex element of disaster recovery. This resource gap underscores the critical part played by community embedded volunteers and nonprofits in supporting affected households through the recovery process. This form of support is a well‐received and valuable initiative: research shows that housing reconstruction and material recovery are preconditions for social recovery post disaster (Tierney and Oliver‐Smith, 2012). Unpacking this impact of volunteers is important for widening research around disaster volunteering and expanding important discussions in disaster management and governance regarding, inter alia, the growing role and strength of the non‐profit sector.

Through an array of support mechanisms, volunteers boost the capacity of individuals and households to respond to, adapt to, and recover from the effects of disasters through relational and individualised support. This is facilitated by empathetic and attentive listening, sustained engagement, and empowering affected people to participate actively in decision making and their own recovery. While Akar et al. (2025) found that volunteers' relational and agile approach benefited teachers and children, we extend this conclusion by showing that these relational qualities are not unique to educational contexts but are fundamental to effective volunteer‐led long‐term disaster recovery more broadly. In many cases, volunteers' contributions enabled housing recovery among those affected by a disaster while also instilling a sense of calm and optimism, leading to the resumption of day‐to‐day household activities. These findings align with existing research suggesting that disaster recovery success is inherently individualistic, hinging on the renewal of regular daily activities (Peacock, Dash, and Zhang, 2007; Sanderson et al., 2025b).

Community connections and local knowledge enable these volunteers to coordinate and collaborate with other service providers in the disaster recovery system. We also note that the capacity of these volunteers to collaborate at the community level to respond to immediate and emerging recovery needs is a sign of resilient local communities. This becomes more important for regional and remote communities that may be isolated after a disaster.

These findings exemplify Aldrich's (2012) theory about the importance of social support in disaster recovery. In practice, volunteers embedded within communities epitomise this social capital. Aldrich (2012) further adds that social ties and support buffer stressors and minimise the outmigration of people and key resources from disaster‐affected regions.

Like Serrao‐Neumann, Crick, and Low Choy (2018), we found that when volunteer‐based recovery agencies engage more deeply with communities, they gain access to invaluable local knowledge and personal networks that enhance the effectiveness of their support services. Volunteers' embeddedness within the local community also provides them with a direct link to vulnerable and marginalised people in the community. Their empathy and dynamic engagement helped them to gain trust and, in turn, support vulnerable people through diverse forms of support needed to enable long‐term recovery. In addition, our findings underline how volunteers support individuals and households throughout the overwhelming and complex recovery process—this is well documented by Greer and Trainor (2021) who showed that the assistance‐seeking process is often characterised by excessive paperwork, adversarial interactions with government personnel, misaligned institutional priorities, and frequently shifting eligibility rules. Our findings are also consistent with prior research that reveals how volunteers provide need‐based support (Mukherji, 2017). This is especially important in relation to vulnerable people and households and those with complex and compounded needs. Our research also uncovers what disaster‐affected communities value: localised, sustained, and relational support.

We note elements of both autonomy and collaboration in the volunteers' work discussed here. Our analysis outlines how volunteers play an integral part rather than supplying residual or secondary assistance, which differs from the complementary roles often discussed in the literature (Akiyama, 2019). This is enabled by professional support in conjunction with the relational approach. Together, these elements create a powerful synergy where much‐needed support is delivered through trusted relationships, making assistance both competent and compassionate.

Furthermore, our findings are consistent with work that reveals how volunteers and volunteer‐based organisations have not only emerged as pivotal actors in social welfare policies, but also are ‘at the forefront of strengthening disaster resilience’ (McLennan, Whittaker, and Handmer, 2016, p. 2032). This will likely be of greater importance going forward, as the Australian *National Strategy for Disaster Resilience* (Council of Australian Governments, 2011) outlined that effective community resilience depends on well‐coordinated relationships among communities, government agencies, and this increasingly influential sector. Consequently, the growing integration of the non‐profit sector and volunteers into formal disaster recovery, management, and risk reduction positions them as intermediaries between affected communities and formal emergency management systems.

## 6 CONCLUSION

Drawing on an Australian qualitative study involving semi‐structured interviews with long‐term volunteers, stakeholders who collaborate with volunteers, and people affected by disasters who received support services, this article contributes to research and practice knowledge by demonstrating how volunteers support individuals and households in recovering from disasters. In relation to our key aim, we identified three interconnected service delivery elements that are particularly effective for disaster recovery. We add to the literature by providing outcomes‐focused research that moves beyond describing what volunteers do to evaluating rigorously how they lead to disaster recovery. Our research reveals that recovery support is effective when it is relational rather than transactional and individualised rather than standardised. Additionally, disaster‐affected people value support that takes a long‐term perspective and leverages local connections and knowledge.

Volunteers are known for their people‐centric support services (Akiyama, 2019). Often, this form of support translates into socio‐emotional support for those vulnerable or at risk. This also relates to sustained support for those affected by disasters, frequently resulting in greater capacity and resilience to cope with the impacts of a disaster in the future. By concentrating on long‐term, formally organised volunteers, this paper examines the sustained contributions of individuals in volunteering roles who maintain ongoing commitments to disaster recovery efforts through formal, established organisational structures, providing insights into the strategic role of volunteer networks in building local community resilience.

Lough et al. (2018) characterised volunteerism as a renewable resource and advocated for greater investment in volunteering infrastructure globally. A critical component of this infrastructural development is recognising the substantial contributions that volunteers make to disaster recovery efforts. In the Australian context, such recognition not only raises awareness but also strengthens volunteers' sense of ownership of and meaningful contribution to their local communities. Our findings underscore the need to integrate volunteering into formal disaster preparedness and management policies. More importantly, we promote valuing volunteer contributions given their inherent worth to the disaster recovery process, rather than merely viewing volunteers as resources to fill gaps in service delivery. This is increasingly important as the growing reliance on volunteers and nonprofits reflects a broader and entrenched pattern of partnership between states and nonprofits in essential service delivery (Wright, Marston, and McDonald, 2011; Salamon and Toepler, 2015). This service delivery partnership has been the norm in Australia, owing to a combination of historical contingencies and a distinct political system (Wright, Marston, and McDonald, 2011). Yet, this partnership underlines a structural dependency; one that places mounting and unsustainable demands on a sector that cannot continue without adequate government support, particularly as volunteering rates decline globally, including in Australia (Chambré, 2020; Innes, Jefferies, and Gates, 2025; Nesbit, Paarlberg, and Jo, 2025). Critically, it is important to recognise that providing disaster recovery support may increase the administrative burden on volunteers (Duffy and Shaefer, 2022) who are already supporting disadvantaged communities through their daily work. This further raises concerns about the sustainability of this model as volunteers continue to provide professional service delivery without adequate recognition or commensurate investment. Together, these factors underscore that the long‐term disaster recovery support provided by volunteers is unsustainable without monetary and non‐monetary support from government.

The effective service delivery approaches identified in our research transcend specific cases and contexts, offering relevance across diverse disaster types and settings. Although not examined directly here, supporting volunteer‐led long‐term recovery may require paying attention to interconnected challenges. These include addressing the rising need for digitally enabled volunteers and service delivery, especially in rural and regional communities affected by disasters (McLennan, Whittaker, and Handmer, 2016), as well as investing in training that builds on the expertise and leadership skills that volunteers cultivate through sustained recovery work. Progress on these fronts, though, may be shaped by the resource constraints that confront volunteers and nonprofits and the coordination challenges posed when working with various service providers (Aldrich, 2019).

Long‐term recovery requires sustained, collaborative, and systematic efforts. It also necessitates financial resources that are often a constraint for volunteers and nonprofits, particularly when they are supporting housing recovery. Collaboration with stakeholders, especially government agencies, is important for attracting financial, informational, and other key resources to sustain these volunteering efforts.

A limitation of this research is the paucity of longitudinal data to grasp fully the dynamic changes and the evolution of the relationship between volunteers and those impacted by a disaster. It is plausible that the disaster‐affected individuals who came forward to participate in this research may have done so in part because of positive experiences of receiving recovery support, and that people with less favourable interactions with volunteers may be under‐represented here. Furthermore, we have insufficient data on the challenges that volunteers face in this role and how the system assists those delivering recovery support to others. Future studies could also extend our empirical knowledge of how formal disaster management agencies recruit, train, and retain volunteers to understand fully the integration of volunteers into broader disaster recovery systems.

## CONFLICT OF INTEREST STATEMENT

The authors declare that they have no competing interests.

## Data Availability

Research data are not shared.

## Endnote

^1^
The Western Downs bushfires constituted one of the largest bushfire events in Queensland. The region also experienced six other disasters triggered by natural hazards between 2021 and 2023: five major floods and one other bushfire (Western Downs Regional Council, 2024). The bushfires particularly impacted communities in and around Tara district and destroyed more than 112 structures. Tropical Cyclone Jasper and associated flooding cost the wider Cairns region around AUD 650 million and even cut off homes in Holloways Beach (Cairns Regional Council, 2024). According to research commissioned by the Queensland Reconstruction Authority, the 2022 floods in southeast Queensland affected more than 500,000 people and resulted in AUD 7.7 billion of social, economic, and health costs (Deloitte Access Economics, 2022). The event impacted approximately 18,000 homes and businesses in the region; 10.9 per cent of them experienced severe damage.
